# Multi-Methodological Quantitative Taste Assessment of Anti-Tuberculosis Drugs to Support the Development of Palatable Paediatric Dosage Forms

**DOI:** 10.3390/pharmaceutics12040369

**Published:** 2020-04-17

**Authors:** Alison V. Keating, Jessica Soto, Claire Forbes, Min Zhao, Duncan Q. M. Craig, Catherine Tuleu

**Affiliations:** 1 UCL School of Pharmacy, 29-39 Brunswick Square, London WC1N 1AX, UK; alison.keating.13@ucl.ac.uk (A.V.K.); jessica.soto@novartis.com (J.S.); duncan.craig@ucl.ac.uk (D.Q.M.C.); 2 Pfizer R&D UK Ltd., Ramsgate Road, Sandwich, Kent CT13 9ND, UK; claire.forbes@pfizer.com; 3 School of Pharmacy, Queen’s University Belfast, Lisburn Road, Belfast BT9 7BL, UK; m.zhao@qub.ac.uk

**Keywords:** tuberculosis, palatability, taste assessment, electronic tongue, biomimetic sensors, human taste panel, BATA model

## Abstract

The unpalatability of antituberculosis drugs is often cited as a major cause of non-adherence in children, yet limited quantitative taste assessment data are available. The aim of this research was to quantify the bitterness of isoniazid, rifampicin, pyrazinamide, and ethambutol dihydrochloride using two in vivo (a human taste panel and a rat brief-access taste aversion (BATA) model) and one in vitro (sensor) method. The response of the Insent TS-5000Z electronic tongue was compared to the in vivo drug concentration found to elicit and suppress half the maximum taste response (EC_50_ in human and IC_50_ in rats). Using dose-relevant concentrations, an overarching rank order of bitterness was derived (rifampicin > ethambutol > pyrazinamid~isoniazid). In vitro, only ethambutol exhibited a linear response for all sensors/concentrations. Based on the EC_50_/IC_50_ generated, a ‘taste index’ was proposed to allow for anticipation of the likelihood of taste issues in practice, taking in account the saturability in the saliva and therapeutic doses; ethambutol and isoniazid were found to be the worst tasting using this measure. The study presents the first quantitative taste analysis of these life-saving drugs and has allowed for a comparison of three methods of obtaining such data. Such information allows the operator to identify and prioritise the drugs requiring taste masking to produce palatable formulations.

## 1. Introduction

Tuberculosis is a major global health problem, ranking alongside HIV as a leading cause of mortality worldwide. In 2016, an estimated 1.67 million people died as a result of tuberculosis infection [1]. The standard, and largely effective, treatment regimen lasts a minimum of six months and requires the patient to take three or four drugs, typically isoniazid, rifampicin, pyrazinamide, and ethambutol dihydrochloride, in combination for two months (intensive phase), followed by rifampicin and isoniazid for four months (continuation phase) [2]. Impediments to successful treatment include the availability of these drugs and patient non-adherence to treatment regimens. There are many reasons for non-adherence to tuberculosis treatment, including the complex and onerous nature of this treatment regimen, an inability to meet the financial burden of treatment, and an inability to complete treatment due to side effects [3]. Another common reason for non-adherence, particularly in paediatric patients, is the refusal to take medicine due to the unpalatability of formulations [4]. There is, therefore, a compelling case for the development of age-appropriate formulations for these drugs; this in turn requires, or would at the very least be facilitated by, a quantitative assessment of the taste of these four frontline drugs that can subsequently be used to assess the taste masking challenges. Despite this recognition, there are a paucity of quantitative data available regarding the bitterness levels of these drugs; this represents a major omission in the knowledge base for tuberculosis treatment and an impediment to the development of paediatric formulations.

These drugs have been reported to be unpalatable, and the bitter taste of these drugs is often cited as a major barrier to treatment adherence, particularly in paediatric patients [5,6,7]. An extensive literature review was carried out to determine what, if any, quantitative or qualitative data were available regarding the taste of these four drugs. However, despite numerous reports of the bitter taste of tuberculosis medicines being a barrier to treatment adherence [4,5,6,7,8], no studies that quantified the bitterness of these drugs were identified. 

To overcome palatability issues, taste-masking and taste assessment are essential steps in the formulation development process. The taste of medicines may be assessed using in vivo or in vitro methods. In vivo methods include human taste panels [9] and animal taste tests such as the rodent brief access taste aversion (BATA) test [10]. In vitro methods [11] include electronic taste sensors, known as electronic tongues [12], and biomimetic dissolution tests [13,14].

Electronic tongue systems are sensor array-based robotic systems that can be used for the assessment of single substances, as well as complex mixtures of substances [15]. These systems can be based on a variety of underlying techniques such as potentiometry, amperometry, voltammetry, or impedance spectroscopy [16,17]. Potentiometric systems are most commonly used for pharmaceutical applications. Potentiometry measures the potential of a solution between two electrodes: a reference electrode that has a constant potential and an indicator electrode whose potential is dependent on the composition of the sample being assessed. There are two commercially available electronic tongues, the Alpha MOS Astree electronic tongue and the Insent TS-5000Z taste sensing system. The sensors employed by these machines attempt to imitate and represent what happens when molecules interact with taste buds in the human oral cavity. When a molecule interacts with a sensor, there is a change in the electrical potential, which typically varies logarithmically with the acuteness of the taste response under examination [18]. A previous study examined the in vitro/in vivo correlations and data reproducibility of the Astree e-tongue and Insent systems, with the latter proven to be more reliable [19].

Animal models, particularly using mice and rats, may also be used to assess taste. The rodent BATA model is an in vivo taste assessment tool that has shown great promise in assessing the taste of active pharmaceutical ingredients (APIs) with comparable results to human taste panel data [10,11,20]. In this animal taste model, rodents are mildly water-deprived and then placed in a ‘lickometer’ that records the number of licks that the rodents make to different concentrations of the API under assessment. A high number of licks indicates that the solution is palatable, whereas a low number of licks compared to water indicates an aversive taste. With this procedure, a full concentration–response curve of lick rate can be obtained over a short period of time with very few animals.

Human taste panel studies involve evaluating the taste of medicines or dosage forms by estimating the gustatory sensation responses in healthy adult volunteers. Human taste panels are generally sensitive measures of taste and are specifically designed to minimise bias and variable responses between the volunteers. However, it should be noted, even with specially trained subjects, it can remain a subjective evaluation method [21].

The overall aim of this study was to investigate how the taste of isoniazid, rifampicin, pyrazinamide, and ethambutol could be meaningfully quantified to support the development of palatable formulations. As these four drugs vary significantly in their properties such as molecular weight, logP, and solubility, this study had the opportunity to provide insight into the key drug properties required for the successful quantification of taste (bitterness/aversiveness) by various methods. In vitro assessment was performed using the Insent TS-5000Z electronic tongue. A human taste panel was used for in vivo taste assessment; this is considered the ‘gold standard’ for taste assessment, but panels are logistically challenging, expensive to run, and, for obvious safety issues, they require ethical approval which increases the time period from inception to completion. The second in vivo test was the rat BATA model, which is quicker and less challenging to run but requires further validation, as it is a relatively new technique. The results of all these studies are presented herein, giving quantitative taste data for this important group of drugs while also furthering the understanding of the most effective means by which to derive such data, with clear implications for the development of taste-masked formulated products.

## 2. Materials and Methods

### 2.1. Materials

Ethambutol dihydrochloride, isoniazid, and pyrazinamide were obtained from Sigma Aldrich (Sigma Aldrich, Dorset, UK). Rifampicin was obtained from Fagron UK Ltd. (Newcastle, UK). Potassium chloride, potassium hydroxide, and tartaric acid were obtained from Sigma Aldrich (Gillingham, UK). Hydrochloric acid was obtained from Fisher Chemicals (Loughborough, UK). All substances were used as received. For human experiments, Good Manufacturing Practice GMP-grade isoniazid, rifampicin, pyrazinamide, and ethambutol dihydrochloride were obtained from Macleods Pharmaceuticals (Mumbai, India). Bottled drinking water (Waitrose Essential, Waitrose Ltd., Bracknell, UK) was used for all human experiments. Deionised water was used for all non-human experiments.

### 2.2. Methods

#### 2.2.1. Preparation of Standard Solutions for Electronic Chemical Sensor Test

A reference solution used for cleaning, and it was prepared by dissolving 30 mmol/L potassium chloride and 0.3 mmol/L tartaric acid in distilled water.

The negatively charged washing solution used for washing the negatively charged sensors (SB2AC0 and SB2AN0) was prepared by diluting absolute ethanol to 30% with distilled water and adding 100 mmol/L hydrochloric acid.

The positively charged washing solution used for washing the positively charged sensors (SB2C00 and SB2AE1) was prepared by diluting absolute ethanol to 30% and adding 100 mmol/L potassium chloride and 10 mmol/L potassium hydroxide.

#### 2.2.2. Measurement Procedure for the Electronic Chemical Sensor Test

All measurements were performed using the TS-5000Z taste sensing system (Insent Inc., Atsugi-shi, Japan) equipped with four lipid membrane sensors and two corresponding reference electrodes (PPM Instruments, West Sussex, UK). Three of the sensors represent bitterness: bitterness sensor 1 (AN0), bitterness sensor 3 (AC0), and bitterness sensor 3 (C00). The fourth sensor represents astringency (AE1). Each measurement cycle consisted of measuring a reference solution (V_r_), followed by the sample solution (V_s_), a short (2 × 3 s) cleaning procedure and measurement of the aftertaste (V_r’_), and finally a 330 s cleaning procedure. The sensor output for taste (relative value, R value) was calculated relative to the preliminary sensor response to the reference solution (V_r_).

(1) $$R={\;V}_{s}-V_{r}$$

The entire measurement procedure was performed for all samples and repeated afterwards up to six times. For data treatment, the first run was discarded (as recommended by Insent) to enable conditioning of sensors.

#### 2.2.3. Taste Solutions for the Electronic Chemical Sensor Test

Solutions of drugs of varying concentrations were prepared in distilled water by dissolving the exact amount of the corresponding API in a fixed volume of water. Sonication (XUBA3 ultrasonic bath, Grant Instruments Ltd., Cambridgeshire, UK) was used to facilitate the dissolution of APIs when required. The concentrations of drugs used are given in Table 1. The pH of each solution was measured using a pH meter (SciQuip 902, SciQuip, Wem, UK), and the ratio of ionised to unionised drug present in solution was calculated using the Henderson–Hasselbalch equation [22] (Equation (2)).

(2) $$\mathrm{pH}=pK_{a}+\log_{10}\left[A^{-}\right]/\left[\mathrm{HA}\right]$$

#### 2.2.4. Data Analysis for the Electronic Chemical Sensor Test

The normality of the data was checked with the Shapiro–Wilk test [23]. As the data were normally distributed, a one-way analysis of variance (ANOVA) was performed followed by post-hoc analysis with Tukey’s honest significant difference test (*p*-value 0.05) [24] to check which concentrations elicited a sensor response that was significantly different to that elicited by deionised water. Dose response curves were created by plotting the mean sensor response (± standard deviation) as a function of concentration. All statistical analyses on electronic tongue data were carried out using OriginPro (OriginLab, Northampton, MA, USA, version 9.0.0).

#### 2.2.5. Taste Solutions for In Vivo Experiments

The drug concentrations used in the present study were chosen based on a number of factors including the water solubility of the drug, the clinical dose of the drug, and rat LD_50_ (Lethal Dose, 50% or median lethal dose) and human acceptable daily intake (ADI) limits. To enable a direct comparison of taste intensity, the concentrations used in both panels were overlapping, but fewer concentrations were tested in humans (four compared to six in rats) to ensure that the risk of taste fatigue was minimised [9]. The concentrations used were well below the level causing no toxicological effects in humans and rats to ensure the safety of both human participants and rats.

Drug solutions were freshly prepared and presented at room temperature. Solutions of varying concentration (Table 1) were prepared in bottled water for the human panels and in deionised water for rats. Sonication (XUBA3 ultrasonic bath, Grant Instruments Ltd., Cambridgeshire, UK) was used to facilitate the dissolution of APIs when required. All extemporaneous preparations were carried out under the supervision of a registered UK pharmacist following standard operating procedures.

#### 2.2.6. BATA Experiment

All procedures were carried out in accordance with the Animals (Scientific Procedures) Act 1986 (Project Licence PPL 70/7668) approved on 29 May 2013. Ten adult male Sprague–Dawley rats (Charles-River, Kent, UK) were used. They were housed in pairs in standard cages in a room that was maintained at 21 ± 2 °C with 55 ± 10% relative humidity and a 12:12 h light/dark cycle. Animals had free access to chow (Harlan, Oxon, UK) and tap water except for training and testing periods, where a water-restriction schedule occurred. Daily food and water consumption were monitored. As a safety and welfare measure, each rat was weighed daily to ensure that their weight did not drop below 85% of their free-feeding weight. A one-week wash-out period was observed for each rat between BATA experiments to ensure that the rats became naïve again and could be re-used.

Each rat was water-deprived for 22 h before each session (training and testing) and then placed in the ‘‘Davis MS-160” lickometer (DiLog Instruments, Tallahassee, FL, USA) for a maximum session-length of 40 min. After each session, the rats were rehydrated in their home cage. The rats received a first training session where the shutter was continuously open, presenting a single tube containing deionised water. In the second training session, they were presented sixteen moving tubes containing deionised water. The training sessions were followed by two testing sessions over 2 consecutive days, during which each rat was presented with either deionised water or one of the six concentrations of drug in a randomised order. One session per day was carried out, and each drug was assessed individually on separate days. Each trial was intercepted by a water rinse to minimise carry over effects. The number of licks was automatically recorded. Each concentration of each compound was trialled four times per session/rat.

#### 2.2.7. Human Taste Assessment

All procedures were carried out in accordance with the UK Data Protection Act 1998 and were approved by UCL Research Ethics Committee (REC) 4612/009 approved on 1/11/2015. Twenty (11 female and 9 male) healthy adults in the age range 18–40 were recruited (median age 26). Individuals with antecedent deterioration of taste or smell, smokers, those who had undergone dental care or medicinal treatment (excluding contraceptives) up to 15 days before the test, or those with known drug allergies were excluded from participating.

A single blind, cross over, single centre study was conducted using the ‘swirl and spit’ method. Four concentrations of each drugs were assessed in triplicate on separate days. A 72 h ‘washout’ period was respected between sessions to minimise the burden on participants. Anonymised samples (10 mL) were presented in a randomised order at 10 min intervals. Participants rinsed their mouths with the solutions for 5 s to cover all oral surfaces before spitting out the sample. Immediately upon expectoration, subjects electronically rated the aversiveness on a 100 mm bipolar scale between 0 (not aversive) and 100 (extremely aversive) (Qualtrics, Version: May–June 2016, Provo, UT, USA). Participants then washed their mouths out with water and ate an unsalted cracker if necessary, to neutralise their palate. All participants tasted 12 samples per session (maximum 2 h).

#### 2.2.8. Data Analysis for the In Vivo Experiments

The normality of all data was checked with the Shapiro–Wilk test [23]. As the data were not normally distributed, the Kruskal–Wallis test [25] was performed followed by a post-hoc analysis that was carried out with Gao et al.’s non-parametric multiple test [26] to check which concentrations were deemed significantly different to water (human taste panel) or elicited a number of licks significantly different to water (BATA model).

The IC_50_ value, which corresponds to the concentration of drug that suppresses 50% of licks, and the EC_50_ value, which corresponds to the concentration of the drug that elicits half the maximum taste response compared to the reference (water), were calculated using the E_max_ model developed by Soto et al. [27]. A 95% confidence interval (CI), variance values for each of the parameters, and error values were obtained from this model as reflections of inter- and intra-participant variability. The variability between the BATA model and human testing was assessed by comparing the standard error of the mean (SEM) for each concentration of drug when assessed by rodents and humans.

Soto et al. [27] also proposed classification for the prediction of the human taste response based on rat BATA data. This was based on 9 APIs and verified with 4 more. An API with an IC_50_ between 0 and 0.1 mM was classified as extremely aversive, between 0.1 and 1 as moderately aversive, between 1 and 10 mM as mildly aversive, and between 10 and 100 mM as weakly aversive. This classification was used to discuss the findings.

All statistical analyses were performed with R (R Core Team, Vienna, Austria, version 3.3.1). OriginPro (OriginLab, Northampton, MA, USA, version 9.0.0) was also used for the generation of any additional graphs. IC_50_ and EC_50_ values were estimated by using the non-linear mixed effects modelling that was performed using the software NONMEM^®^ (ICON, Ellicott City, Maryland, version 7.3) in conjunction with a gfortran (64-bit) compiler using Perl-Speaks NONMEM ^®^ (PSN, version 4.2.0) as an interface to run NONMEM^®^.

## 3. Results

### 3.1. Ethambutol Dihydrochloride

**Figure 1:**
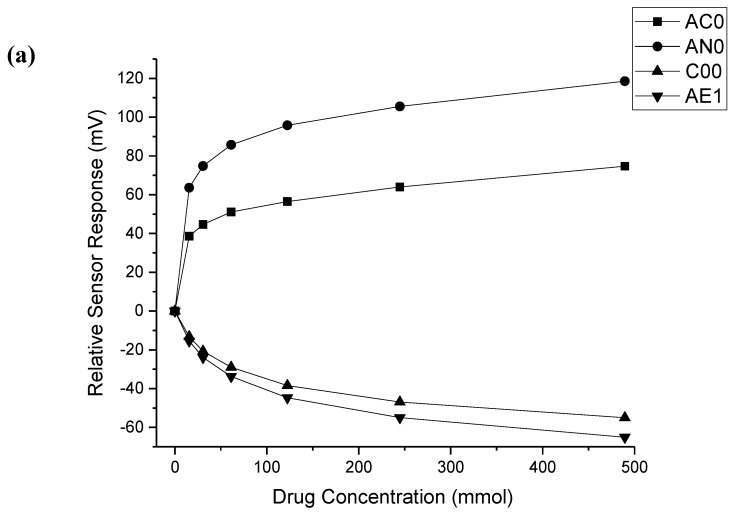

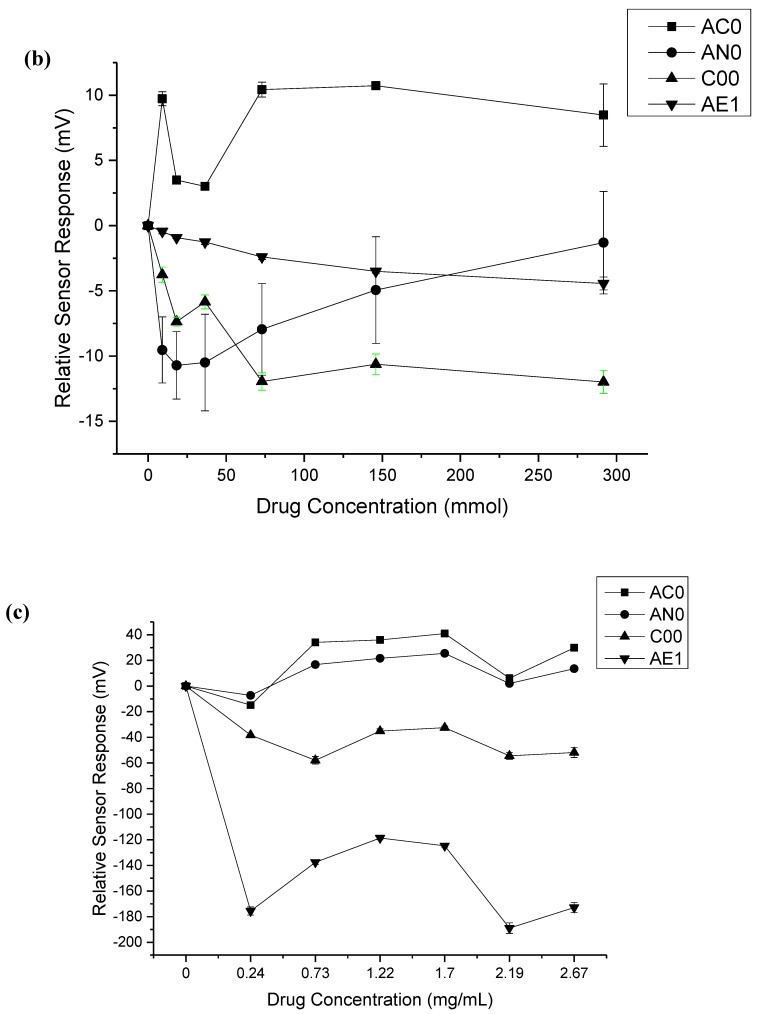

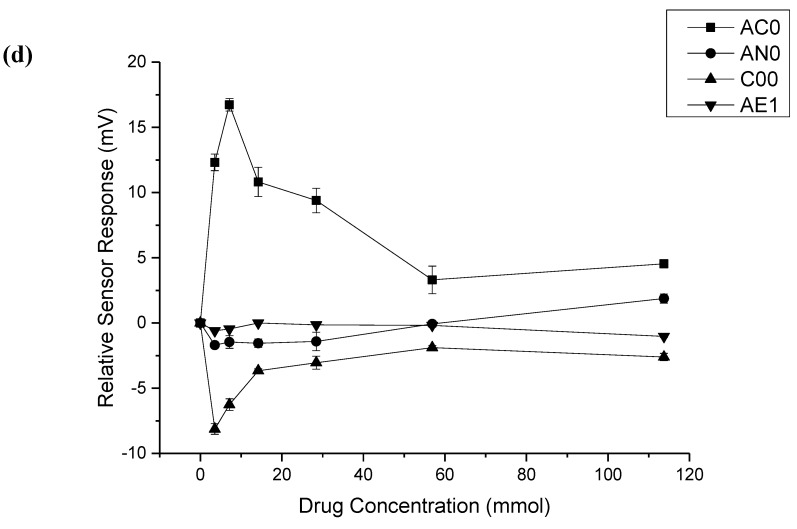
Sensor response curve for (**a**) ethambutol dihydrochloride, (**b**) isoniazid, (**c**) rifampicin, and (**d**) pyrazinamide showing normalised sensor response as a function of concentration (*n* = 6, mean ± S.D.).

The responses of all four sensors to ethambutol dihydrochloride are given in Figure 1a. All concentrations of ethambutol dihydrochloride were deemed significantly different from water and each other by the four sensors (*p* < 0.05); a clear linear dose response was seen for each sensor. As it is a chloride salt, this drug was ionised under the test conditions, and, hence, the interaction with the lipid membrane of the sensors was facilitated, thus eliciting a clear dose response.

### 3.2. Isoniazid

The responses of all four sensors to isoniazid are given in Figure 1b. Only the C00 sensor was capable of differentiating all concentrations of isoniazid from water (Table S1). It might be expected that if a sensor were to be unable to differentiate between water and a drug solution, it would be the lowest concentrations that are indistinguishable; however this was not the case for isoniazid. The AC0 sensor could distinguish the 18.23 and 36.46 mM solutions from water, whereas AN0 could not distinguish the two highest concentrations from water. The AE1 sensor could not distinguish the three lowest concentrations of drug from each other or water; however this sensor detects astringency rather than bitterness, so it is possible that the three lowest concentrations of drug solution were not significantly more or less astringent than water or each other.

The sensors were also not able to clearly distinguish varying concentrations of drug from each other. For example, all except the C00 sensor were capable of distinguishing the two highest concentrations of drug from each other, which may have been due to the saturation of the sensors. However, in other cases, the sensors were unable to distinguish seemingly random concentrations of drug, e.g., the AC0 sensor could not distinguish 9.11 from 72.92 mM isoniazid, despite the close to order of magnitude difference between these concentrations.

**Figure 2:**
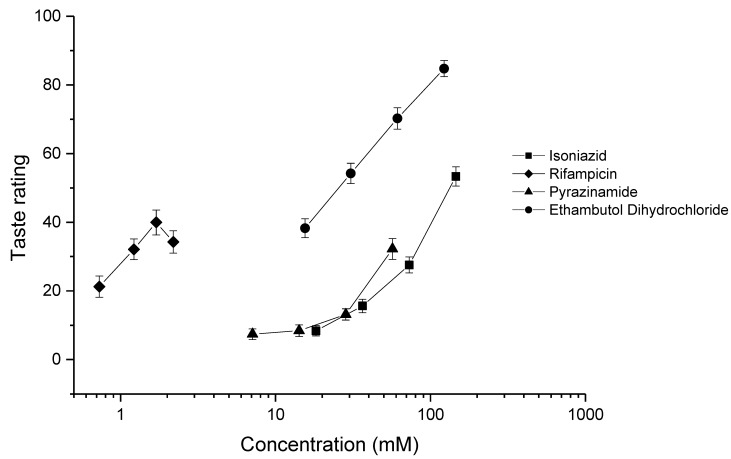
Average taste ratings (± SEM) as a function of concentration for isoniazid [31], rifampicin, pyrazinamide, and ethambutol dihydrochloride (*n* = 20 participants).

As alluded to above, drug molecules more easily interact with the lipid membranes of the sensors when they are ionised. The pH of all solutions of isoniazid was measured as 6.85. Isoniazid has three pK_a_ values (Figure 2), the hydrazine nitrogen has a pK_a_ of 1.8, the pyridine nitrogen has a pK_a_ of 3.5, and the deprotonation of the hydrazide group to a mesomerism stabilised anion has been reported to have a pK_a_ of 10.8 (all measured at 25 °C) [28]. The percentage ionised of each ionisable group was calculated at pH 6.85; these values are given in Table 2. Overall, less than 0.05% of the drug was ionised at this pH, thus making it less easy for the drug molecules to interact with the lipid membrane.

### 3.3. Rifampicin

The sensor responses of all four sensors to rifampicin are given in Figure 1c. In the case of rifampicin, it was only the AN0 sensor that could not distinguish all concentrations of drug from water (Table S2). Similar to what was seen for isoniazid, it was not the lowest concentration of drug that the sensor could not distinguish from water. For the AN0 sensor, the 2.19 mM solution was not deemed significantly different to water. The sensors were also unable to distinguish between certain concentrations of rifampicin, e.g., the C00 sensor was unable to distinguish between 0.73 and 2.19 mM solutions of rifampicin.

Rifampicin is amphoteric and thus has two pK_a_ values: The 4-hydroxyl group has a pK_a_ of 1.7, while the 3-piperazine nitrogen has a pK_a_ of 7.9. It has an isoelectric point at pH 4.8 in aqueous solution [29]. The pH of the rifampicin solutions was measured to determine if the compound was likely to be ionised. The pH of all solutions was measured and found to decrease from 6.02 for the 0.24 mM solution and to 5.75 for the 2.67 mM solution. The percentage ionised of each ionisable group was calculated at these pH values and was found to be 98–99% ionised for each group at all concentrations. This suggests that the drug should interact well with the charged lipid membrane. Looking more closely at Figure 1c, it can be seen that from 0.24 to 1.7 mM, a reasonably linear response was observed for each sensor. At concentrations above this, the response becmae erratic, possibly due to the saturation of the sensor. Another possibility to consider is that while each ionisable group was found to be ionised under the experimental conditions, they had opposite charges. The 4-hydroxyl group lost a proton to become negatively charged, and the 3-piperazine nitrogen gained a proton to become positively charged. It is possible that these opposing charges largely cancelled each other out, which may account for the erratic response observed from the sensors.

### 3.4. Pyrazinamide

The responses of all four sensors to pyrazinamide are given in Figure 1d. Both the AC0 and C00 sensors were capable of differentiating all concentrations of pyrazinamide from water (Table S3). Again, it can be seen that it was seemingly random concentrations of pyrazinamide that could not be distinguished from water, with the 56.89 mM solution and the 14.21 mM solution not being recognised by the AN0 and AE1 sensors, respectively. In terms of differentiating between the solutions of drugs themselves, the AN0 sensor, in particular, struggled to distinguish between individual concentrations of drug. Notably, none of the sensors were able to differentiate 14.21 and 28.43 mM solutions of pyrazinamide from each other.

Pyrazinamide is a very weak base that has been reported to have a pK_a_ value of 0.5 [30], so it is extremely unlikely that the drug was protonated under the test conditions, therefore making it challenging for the drug to interact with the lipid membrane of the sensors.

### 3.5. Human Taste Panel

The results of the human taste ratings of the four drugs are given in Figure 2. The taste intensities of each concentration of drug were calculated for each participant by calculating the mean of the ratings (*n* = 60) obtained from the 20 volunteers. For isoniazid [31] and ethambutol dihydrochloride, all concentrations were found to be less palatable than water. For rifampicin, all concentrations were less palatable than water; however, the higher concentrations (1.22–2.19 mM) were not significantly different to each other (*p* = 0.1821, 0.2993, and 0.2993). Finally, for pyrazinamide, all concentrations were found to be less palatable than water. However, the two lower concentrations (7.11 and 14.21 mM) were not found to be significantly different to each other (*p* = 0.3505).

The data obtained were fitted to the E_max_ model [32], which allowed for the calculation of EC_50_ and 95% confidence interval values, as well as inter- and intra-participant variability (Table 3). After ranking the drugs using the EC_50_ values, it was found that rifampicin > ethambutol dihydrochloride > pyrazinamide > isoniazid, rifampicin being clearly much more aversive than the others (Figure 2). It should be noted that, since the maximum taste response was not achieved for isoniazid, rifampicin, and pyrazinamide, the model extrapolated to determine the EC_50_; therefore, for these drugs, the EC_50_ was estimated. For rifampicin, the maximum concentration of the drug used was limited by the low water solubility of the drug (approximately 0.85 mg/mL). It would be impossible to use the concentrations of rifampicin required to achieve maximum taste response without adding a solubilising agent or changing the pH of the solution, which, in turn, could affect the taste. For isoniazid and pyrazinamide, increasing the concentrations to the values required to achieve the maximum taste response would require giving very large doses of drugs to the participants, which could cause unwanted side-effects. While the human EC_50_ values were estimates, they were still useful guidelines as to the taste intensity of the drugs and will be useful for future formulation development. The EC_50_ values obtained were compared to those of known aversive compounds (quinine hydrochloride and caffeine citrate); this is described in Section 3.8.

### 3.6. Correlation between Human Taste Panel Scores and Electronic Tongue

**Figure 3:**
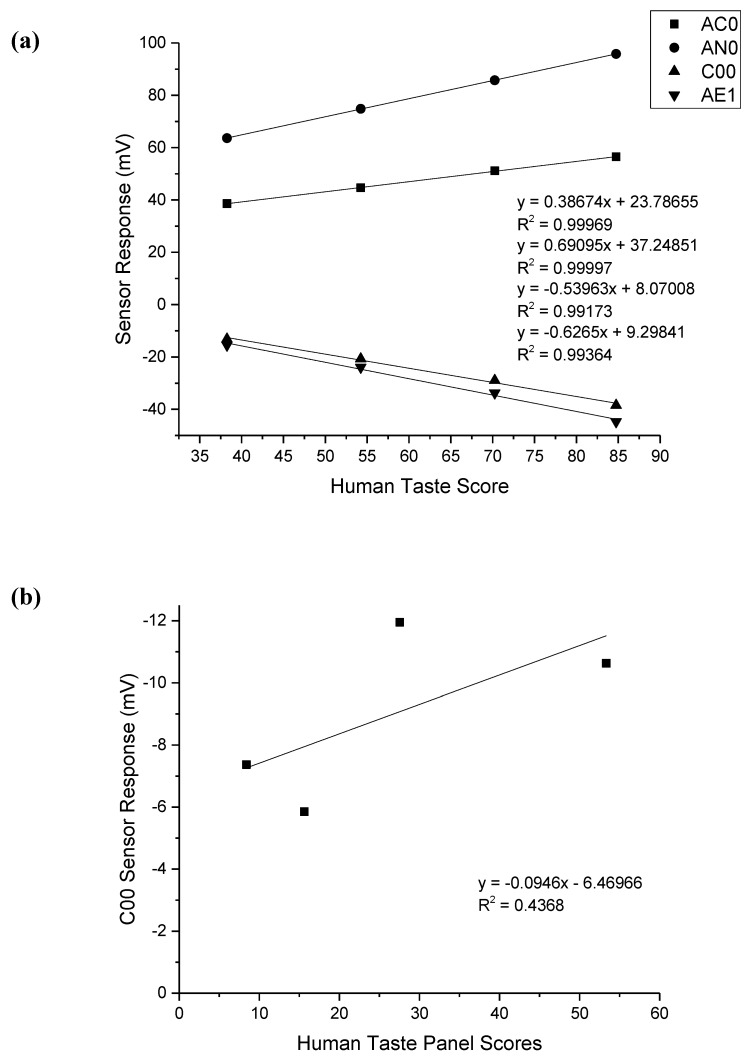

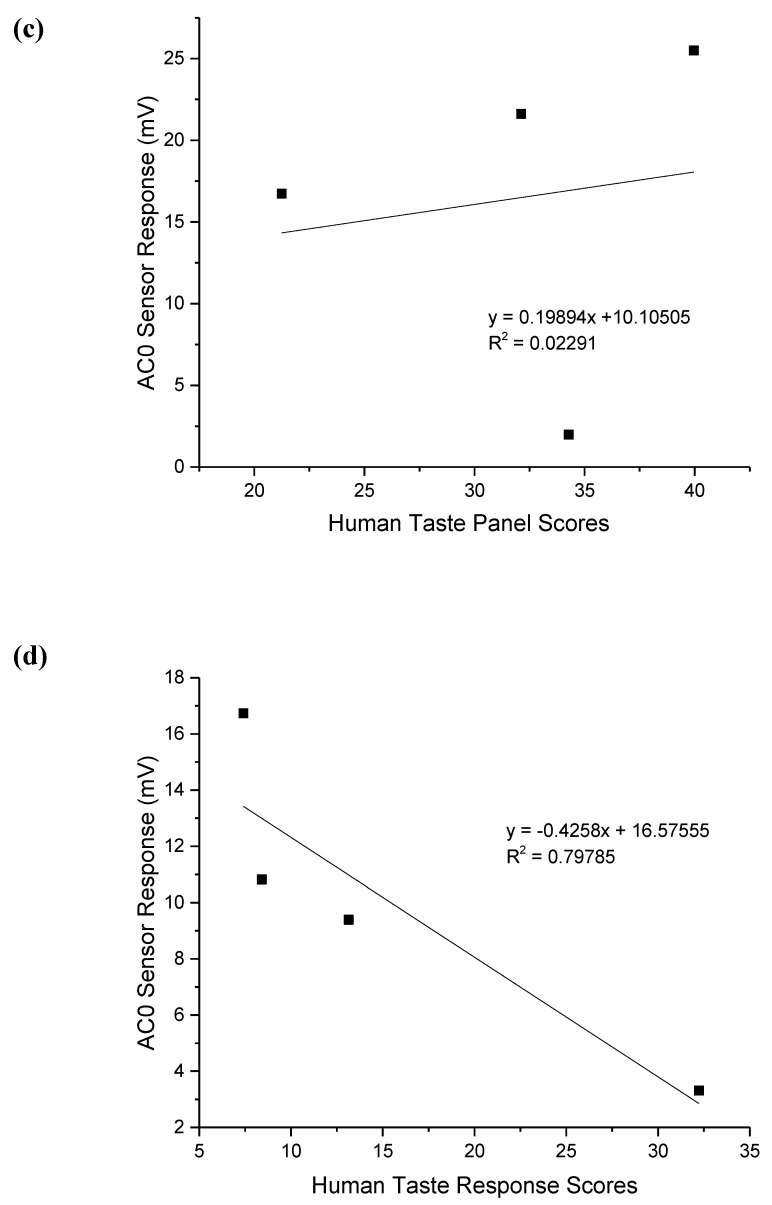
Correlation between human taste scores and sensor responses for (**a**) ethambutol dihydrochloride (all sensors), (**b**) isoniazid (C00 sensor), (**c**) rifampicin (AC0 sensor), and (**d**) pyrazinamide (AC0 sensor).

The correlation between human taste panel scores and the electronic tongue response for each drug was assessed by choosing the individual sensor for which the most linear response was observed and comparing these values to human taste panel scores. For isoniazid, the C00 sensor was chosen, while the AC0 sensor was used for both rifampicin and pyrazinamide. For ethambutol dihydrochloride, since all sensors responded in a linear fashion, the response of all four sensors was compared to human taste panel scores. The results of this are shown in Figure 3.

The correlation between sensor responses and human taste scores for ethambutol was very high, with all sensors having a correlation coefficient in excess of 0.99. The highest correlation for ethambutol was observed for the AN0 sensor with an R^2^ value of 0.99997. It can be seen that for isoniazid and rifampicin, in particular, that the correlation between responses was quite low at 0.4368 and 0.02291, respectively. For pyrazinamide, the correlation was higher at 0.79785.

### 3.7. BATA Testing

**Figure 4:**
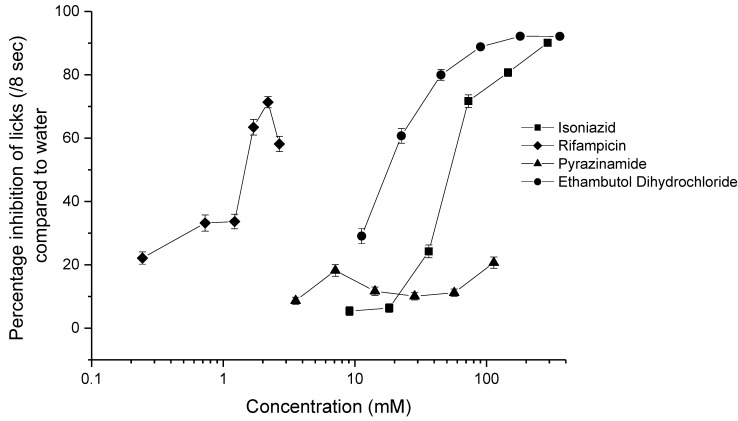
Percentage inhibition of licks as a function of concentration (mM) for isoniazid, rifampicin, pyrazinamide, and ethambutol dihydrochloride (*n* = 10 rats).

The results of BATA testing of the four drugs are given in Figure 4. Isoniazid, rifampicin, and ethambutol dihydrochloride showed a clear aversive taste that could be quantified by an IC_50_ value. None of the concentrations tested for either ethambutol dihydrochloride or rifampicin were as palatable as water. For isoniazid, all except the two lowest concentrations (9.11 and 18.23 mM) were statistically different from water (*p* = 0.53 and *p* = 0.41). However, for pyrazinamide, only the highest concentration, 113.72 mM, had a significantly different number of licks compared to water (*p* = 0.0013), yet the number of licks was still high at 43.15 ± 14.71 licks/8 sec for 113.72 mM pyrazinamide versus 49.07 ± 9.55 licks for water. This indicated that the drug was not aversive at the concentrations tested for the rat taste panels and, thus, was the most palatable agent among the four under assessment.

As each drug was assessed over the course of two days, the interday variability was assessed to determine if there was a significant difference between the results on Day 1 and Day 2. The only concentration of drug for which a significant interday variability was observed was for 3.55 mM pyrazinamide (*p* = 0.002), which was rated more aversive on Day 2 than on Day 1.

IC_50_ values (Table 4) were calculated for isoniazid, rifampicin, and ethambutol using an E_max_ model [27]. Since all except the highest concentration of pyrazinamide were not found to be significantly different to water by the rats, an IC_50_ value could not be determined for this drug, but the flat profile of the curve anticipates that the value would be relatively high.

After ranking the drugs using the IC_50_ values and curve profiles, it is found that rifampicin > ethambutol dihydrochloride > isoniazid > pyrazinamide* (*based on curve profile but not on IC_50_), rifampicin again being ranked significantly more aversive than the drugs.

### 3.8. Comparison of Human Taste Panel and BATA Experiments

The EC_50_ and IC_50_ values indicated that both humans and rats ranked rifampicin as the most aversive API, followed by ethambutol dihydrochloride. Isoniazid was ranked as the third most aversive drug by rats, while humans ranked it as the least aversive of the four drugs. Conversely, rats were relatively insensitive to the taste of pyrazinamide, whereas humans found pyrazinamide to be somewhat aversive, with each sample being deemed significantly different to water. For isoniazid, rifampicin, and ethambutol dihydrochloride, rats were found to be more sensitive to the taste of the API compared to humans, while humans were more sensitive to the taste of pyrazinamide. Participants were able to distinguish even the lowest concentration as significantly different to water, while in BATA studies, only the highest concentration of pyrazinamide was deemed significantly different to water. For this reason, an IC_50_ value could not be obtained for pyrazinamide from BATA experiments.

The log(IC_50_) values and log(EC_50_) values are compared in Table 5. For three of the drugs, the values obtained from rats and humans were within one-half log unit of molar concentration of each other, which is in line with the classification proposed by Soto et al. [31]. The log(IC_50_) and log(EC_50_) values for quinine hydrochloride dihydrate (representing a strongly aversive compound) and caffeine citrate (representing a mildly aversive compound) are included for comparison [31].

It is interesting to note that for rifampicin in both rats and humans, the plateauing of responses occurred at the highest concentrations. For human taste sensation, the Weber–Fechner law states that the threshold of discrimination between two stimuli increases logarithmically with the intensity of the stimulus [33]. In practice, this means that upon reaching a certain threshold value, differences in taste intensity can no longer be detected, which may be what happened in the case of rifampicin. Alternatively, it may be that as the concentrations of drug used were too close to each other to allow for the full differentiation between samples. Unfortunately, due to the solubility limitation with rifampicin, a larger concentration range could not be used. Nonetheless, it was ranked the most aversive of the four drugs.

**Figure 5:**
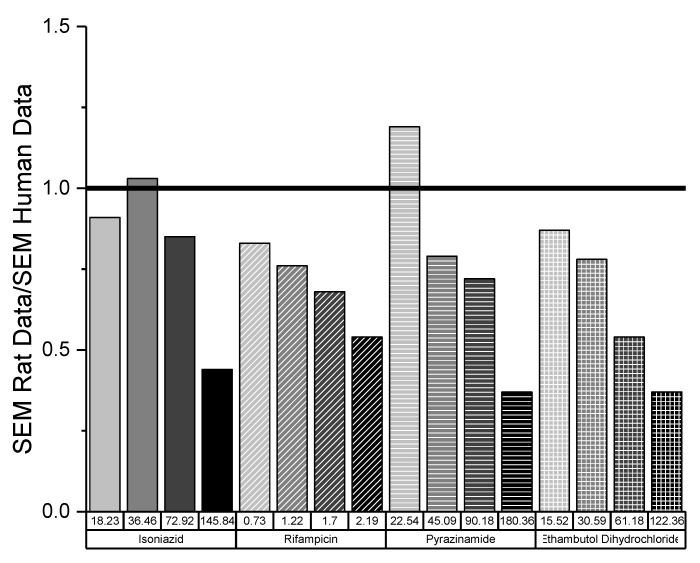
Ratio of SEM for rat and human taste data.

The variability in responses from both taste panels was assessed by calculating the ratio of the standard error of the mean (SEM) for each concentration of drug in rats and humans (Figure 5). In general, the SEM of the rat data was lower than that of the human data, as indicated by the ratio of SEMs being less than 1. Only on two occasions was the SEM of human data slightly above SEM of rat data, demonstrating that overall the variability was lower in rats than in humans. It is also interesting to note that as the concentration of drug increased, the ratio tended to decrease, with the lowest variability between rat and human responses being observed for the highest concentration of each drug. This suggests that as the aversiveness of solutions increased, the variability in taste perception decreased.

### 3.9. Determination of Taste Index

Overall, based on the obtained IC_50_ and EC_50_ values, these drugs could be classified as mildly to weakly aversive, as the values were similar or larger than those of caffeine citrate, which is known to be mildly aversive [31]. However, when considering the taste of drugs, it is not only the IC_50_/EC_50_ values that are important but also the solubility in saliva and the dose administered.

In exploring the relationship between the physicochemical properties of paediatric drugs of various Biopharmaceutical Classification System (BCS) class drugs and their bitter baste sensor response, the dose number (D0) was used by Haraguchi et al. [34]. This took not only the solubility of the drug but also the highest dose taken with a 250 mL glass of water in account. However, paediatric water volumes taken with a dose are rarely that large [35]. Moreover liquid dosage forms would be administered without any water. Hence, the authors propose a slightly different approach herein.

A drug must be in solution in the mouth in order for it to interact with the taste receptors on taste cells and buds and, thus, elicit a taste response. Therefore, the solubility of the drug is of prime importance, as it will determine, from the dose, the maximum concentration of drug that can dissolve/dilute in the saliva (1–3 ml). To determine how problematic the taste of a given drug is likely to be, these parameters, as well as the EC_50_ or IC_50_ values, need to be concomitantly considered. If the saturated solubility of a drug is significantly higher than the EC_50_/IC_50_ and the dose is relatively high, then the taste of the drug will be particularly problematic, as these values will be easily exceeded within the oral cavity.

We propose the use of a taste index (TI) to allow the IC_50_/EC_50_ values to be contextualised. This index can be calculated by dividing the aqueous solubility of the drug by its EC_50_ or IC_50_ and multiplying it by a saliva saturability index, i.e., the quantity of drug divided by the quantity of drug that can dissolve in saliva. The dose administered and the volume of saliva present in the mouth dictates the effective drug concentration so that the highest dose should be divided by the drug quantity that can dissolve in saliva to find out the maximum amount of drug in solution. Presently, we used the amount of drug dissolved in water, not saliva, as a proxy to calculate the TI, and we considered that 1 mL of saliva was available in the mouth. For tuberculosis or TB drugs, it is to be noted that doses are quite high (over 100 mg/dose). The saliva saturability index has to be capped to one when the dose is larger than the solubility. Therefore, the TIs for each TB drug presented (Table 6) were determined using the following equation:

(3) $$\mathrm{TI}=\frac{\mathrm{Saturated}\;\mathrm{Solubility}\;\mathrm{of}\;\mathrm{Drug}\;\times \;\mathrm{Saliva}\;\mathrm{Saturability}\;\mathrm{Index}}{{\mathrm{EC}}_{50}{\;\mathrm{or}\;\mathrm{IC}}_{50}}$$

It can be seen that ethambutol dihydrochloride had the highest TI (calculated with both IC_50_ and EC_50_), even with a 40% saliva saturability index for this highly soluble hydrochloride salt; this high TI value reflects the concomitant low EC_50_/IC_50_. Of the four drugs, this will be the most problematic in terms of taste, as the concentration of a saturated solution will be approximately 72 times the EC_50_ and 106 times the IC_50_. Similarly, isoniazid is also likely to be problematic, as a saturated solution will be approximately 3.5 times the EC_50_ and 11.3 times the IC_50_. Conversely, although rifampicin has the lowest EC_50_ and is therefore objectively the most bitter, it has the lowest TI due to its low aqueous solubility. Pyrazinamide also has a low TI due to its relatively low aqueous solubility.

The higher the TI, the more problematic the taste of the drug is likely to be. It is proposed that a TI below 1 should not raise much concern and that a TI above 10 will require taste masking. Further investigations are ongoing to verify this hypothesis. Nevertheless, the TI is especially useful because it can be calculated with either the IC_50_ or EC_50_, depending on which data the formulator have available to them.

## 4. Discussion

There is a critical need to improve TB treatments for children. Recently Dr. Mel Spiegelman, President and CEO of the TB Alliance, stated: “No child should die of TB. The poor taste of drugs, particularly of multi drug resistant MDR-TB treatments, which often need to be taken for longer than a year, is a critical issue to tackle to improve treatment for children.” [40] The development of age-appropriate, taste-masked formulations is essential to address this; however there are a paucity of quantitative data regarding the bitterness of these drugs. The lack of such data is a significant limiting factor in the development of taste-masked formulations for the treatment of paediatric tuberculosis.

The present study addresses this issue using both in vitro and in vivo approaches, thereby not only supplying quantitative data for the four drugs but also allowing for further comparison and validation of the respective techniques. This work facilitates the comparison of these drugs with other/novel TB drugs to allow for potential palatability issues to be anticipated early in the formulation development process. These quantitative data can also feed into interdisciplinary basic research on taste, e.g., into the development of cell-based tools that are often used to understand the development and function of chemosensory function.

The assessment of isoniazid, rifampicin, and pyrazinamide using the Insent TS-5000Z electronic tongue proved challenging. The factors that can affect the detection of a drug by the taste sensor are: (i) the interaction between detecting sensors and ions, (ii) the extent of dissociation/ionisation of electrolyte, (iii) the concentration of the drug, and (iv) the effect of the solvent [41]. Each sensor membrane is composed of a different artificial lipid and plasticiser that are designed to detect different taste attributes, i.e., sweet, salty, sour, sweet, astringent, or bitter. In the present study, four types of bitterness sensors, AC0, AN0, C00, and an astringency sensor (AE1) were fitted; the bitter sensors represent basic bitterness, neutral bitterness, acidic bitterness, and the bitterness of hydrochloride salts, respectively. The acidic bitterness and astringency sensors have positively charged membranes, and the basic bitterness sensors have negatively charged membranes. As the membranes are charged, it can be assumed that the drug must dissociate and be in its ionised form in order to elicit a change in membrane potential. Strong electrolytes that are completely ionised or dissociated, e.g., ethambutol in this study, have more ions in solution that can interact with the electronic tongue. This is in contrast to the human tongue, which is also capable of detecting dissolved molecules that are not ionised. The field of in vitro taste assessment is continually evolving. For the Insent etongue, a new taste sensor BT0 has been introduced but was not available at the time of our study; it was designed to be more useful for evaluating the bitterness of hydrochloride salt compounds [34]. Moreover, in recent years, bioelectronic tongues (BioETs), integrating biological materials and various types of transducers, have been proposed to bridge this gap between chemical sensors and biological taste [42].

The Henderson–Hasselbalch equation was used in this study to calculate the degree of ionisation of the drugs being assessed. Previous work has indicated that when a drug is unionised, the molecule will not be detected by the taste sensor. Gondongwe investigated the dose response of a variety of drugs including theophylline, theobromine, caffeine, caffeine citrate, and quinine [41]. It was found that drugs such as caffeine, theophylline, and theobromine, which were unionised under the experimental conditions, showed no dose response, while caffeine citrate and quinine, which were ionised, showed a linear dose response. Uchida et al. [43]. also reported the same phenomenon with caffeine and theophylline not eliciting a response from the electronic tongue, despite being classed as aversive in human sensory panels.

In the current study, ethambutol dihydrochloride was the only drug to be fully ionised and to show a true linear dose response, thus allowing the molecule to interact easily with the lipid sensors. According to the Henderson–Hasselbalch equation, isoniazid and pyrazinamide would not be ionised under the experimental conditions; however, they did elicit a sensor response (albeit an erratic one), which is contrary to the findings reported by Uchida et al. and Gondongwe. Ito et al. demonstrated that a potentiometric response may be elicited from lipid membranes by neutral molecules under certain conditions. If a molecule is sufficiently lipophilic, it may partition into the membrane and then dissociate, releasing an ion into the aqueous phase and generating a potentiometric response [44]. This is unlikely to be what is happening in the case of isoniazid and pyrazinamide, however, as these molecules are not lipophilic, as evidenced by their low logP values of −0.64 [45] and −1.884 [46], respectively. Further work will need to be done to investigate how these drugs interact with the lipidic membranes of the sensors. High concentrations of rifampicin elicited erratic responses too. This may have been due to the fact that upon reaching a certain threshold value, differences in taste intensity could no longer be detected, or, alternatively, the concentrations of drug used were too close to each other to allow for the full differentiation between samples. Rifampicin is a lipophilic molecule with a logP of 3.719 [47]; thus, it is likely that the drug is partitioning into the lipid membrane of the sensor affecting the sensor response. This may also contribute to the erratic results observed at higher concentrations.

The results above, combined with the results of Gondongwe [41] and Uchida et al. [43], demonstrate that it is essential to first assess the dose response of the drug being analysed before assessing any formulations containing the drug: Drugs exhibiting a linear dose response are ideal, while those that produce no response must be assessed via different means than electronic tongue. Drugs that exhibit an erratic dose response, e.g., isoniazid or pyrazinamide, could still be assessed using the electronic tongue only if the quantity of drug being assessed exhibits a significantly different response to that of water. Haraguchi et al. tested isoniazid (and another 46 paediatric drugs) at a 0.1 mM (albeit 10 times smaller than our lowest concentration presently tested). Its bitterness, as per the same Insent, taste sensors (AC0, AN0, BT0, C00, and AE1) was ranked: It belonged to group 1 of drugs with caffeine. Quinine was at the opposite end of the spectrum in group 4. Isoniazid was the only TB drug tested, but this result was in line with the work of Soto et al., where it was classified as mildly aversive (like caffeine) [31].

The correlation between human taste panel responses and electronic tongue sensor responses for each drug was compared. A poor correlation was observed for isoniazid and rifampicin, while a reasonable correlation was observed for pyrazinamide. An excellent correlation was observed between human and electronic tongue responses for ethambutol dihydrochloride, with all sensors having an R^2^ value in excess of 0.99. It is unsurprising that ethambutol dihydrochloride had the highest correlation coefficient, given that it was the only drug which exhibited a fully linear response from each sensor; however it was both surprising and encouraging that the correlation was so good.

The assessment of the drugs by human taste panel allowed for the calculation of EC_50_ values (Table 3). Comparing the EC_50_ values to that of quinine, a known strongly bitter compound (EC_50_: 0.257 mM) [32], these drugs would be considered mildly to weakly bitter. However, the aversiveness of a drug cannot be defined by EC_50_ alone: The solubility and dose of the drug are also important factors. On this basis, a ‘taste index (TI)’ was proposed. It compares the EC_50_ to the aqueous solubility and amount of drug that can solubilise in the saliva and elicit a taste response; the higher the TI of a drug, the more problematic the taste is likely to be. The TI will be a useful tool for formulation scientists to rapidly determine how aversive the taste of a drug is likely to be. In this study, the most problematic drugs in terms of taste were isoniazid and ethambutol. It is important to note that the aversive taste response is likely to be even more acute in paediatric patients, as it has been shown that children are more sensitive to the bitter taste of drugs than adults [48]. Thus, it is essential that paediatric formulations of these drugs, particularly those containing ethambutol and isoniazid (as indicated by their high TI) are developed with appropriate taste masking to improve adherence rates.

The four drugs were also assessed using the rodent BATA model, which allowed for the calculation of IC_50_ values for isoniazid, rifampicin, and ethambutol. The IC_50_ values obtained from BATA testing were lower than the EC_50_ values obtained from human testing, indicating that rats are more sensitive to the taste of these medicines than healthy adults. Given that children are more sensitive to the bitter taste of medicines than adults are [48], this suggests the BATA model could especially useful as a model to calculate the TI in order to formulate age-palatable paediatric medicines. In all cases, the IC_50_ and EC_50_ values obtained were within one half-log unit of molar concentration of each other, which is in accordance with previous studies of this type [10,20,31,32]. The BATA model is showing great promise as a tool to assess the aversiveness of APIs and is gaining interest within the pharmaceutical industry [49]. It is possible that, in the future, its use could be expanded to assess the taste of formulations [50,51], in which case the BATA data obtained in this study would be extremely useful to facilitate the formulation development of these drugs.

A larger variability was observed in human responses compared to those of the rats. This was most likely due to different perceptions of taste that can be as a result of many factors including gender, age, diet, culture, and genetics [48,52,53,54]. While the human participants came from a variety of backgrounds and cultures, with associated differences in taste perception, the rats used for BATA testing were much more uniform in terms of environment and diet, which may explain their lower variability. Another factor to consider is that panellists were not screened for bitterness sensitivity before being selected for the study.

## 5. Conclusions

While the unpalatability of antituberculosis drugs is often cited as a major cause of non-adherence in children, no quantitative reports on their taste assessment are available. The aim of this research was to quantify the bitterness of isoniazid, rifampicin, pyrazinamide, and ethambutol dihydrochloride using two in vivo (a human taste panel and a rat brief-access taste aversion (BATA) model) and one in vitro (biosensor) method. We explored the use of the Insent TS-5000Z electronic tongue to measure bitterness, while the in vivo approaches allowed for the determination of a drug concentration that elicited and suppressed half the maximum taste response (EC_50_ in human and IC_50_ in rats). In vitro, only ethambutol exhibited a linear response for all sensors/concentrations. This was attributed to different drug ionisation states. In vivo, an overarching rank order was derived (rifampicin > ethambutol > pyrazinamide/isoniazid) using dose-relevant concentrations, with the EC_50_ and IC_50_ values being within one half-log unit of each other. However, given the variations in both the solubility and required dose for each drug, a taste index (TI) was proposed to allow for the likelihood of taste issues manifesting in practice to be identified. That way, ethambutol dihydrochloride and isoniazid (as indicated by their higher TI values) could be identified as the ones to be developed with appropriate taste masking to produce more palatable formulations and ease adherence issues. Overall, this study provides the first direct comparison of three taste assessment methods for the four first line TB drugs and also provides the first quantitative taste analysis of this important group of drugs. It also proposed how to identify drugs requiring the most taste masking to produce palatable formulations.

## Figures and Tables

**Table 1:** Concentrations of drugs used for taste assessment. All concentrations were assessed using the brief-access taste aversion (BATA) model and an e-tongue.

Drug	Concentrations (mM)					
Ethambutol dihydrochloride	15.52 *	30.59 *	61.18 *	122.36 *	244.73	489.45
Isoniazid	9.11	18.23 *	36.46 *	72.92 *	145.84 *	291.67
Rifampicin	0.24	0.73 *	1.22 *	1.70 *	2.19 *	2.67
Pyrazinamide	3.55	7.11 *	14.21 *	28.43 *	56.89 *	113.72

* shows that only 4 out of 6 concentrations for each drug were assessed by the human taste panel.

**Table 2:** Percentage of each group ionised at pH 6.85 based on known pK_a_ values of isoniazid.

Group	pK_a_	Percentage Ionised
Hydrazine nitrogen	1.8	0.0009%
Pyridine nitrogen	3.5	0.0440%
Hydrazide	10.8	0.0110%

**Table 3:** EC_50_ (concentration of the drug that elicits half the maximum taste response compared to the reference (water)), 95% confidence interval, and inter-subject and intra-subject variability obtained from the E_max_ model.

Drug	EC_50_ (mM)	95% Confidence Interval	Inter-Subject Variability	Intra-Subject Variability
Isoniazid [31]	259 *	80.0–437.9	0.843	95.7
Rifampicin	3.6 *	1.2–6.0	1.36	211
Pyrazinamide	158 *	90.8–225.2	0.336	161
Ethambutol dihydrochloride	27	18.8–35.2	0.335	227

* estimated EC_50_ value.

**Table 4:** IC_50_ (the concentration of drug that suppresses 50% of licks) values obtained for isoniazid, rifampicin, pyrazinamide, and ethambutol calculated from BATA testing results using the E_max_ model [27].

Drug	IC_50_ Value (mM)
Isoniazid	80.94
Rifampicin	1.31
Pyrazinamide	Could not be calculated
Ethambutol dihydrochloride	13.63

**Table 5:** Comparison of rat IC_50_ and human EC_50_ values obtained from fitting of data to the E_max_ model.

Drug	log(IC_50_)	log(EC_50_)	Log Difference
Isoniazid [31]	1.91	2.41	0.50
Rifampicin	0.12	0.56	0.44
Pyrazinamide	-	2.20	-
Ethambutol dihydrochloride	1.13	1.43	0.30
Quinine hydrochloride dihydrate [31]	−1.10	−0.59	−0.51
Caffeine citrate [31]	0.89	0.70	0.19

**Table 6:** Aqueous solubility, EC_50_ values, IC_50_ values (converted in mg/mL), and respective taste indices (TI) for isoniazid, rifampicin, pyrazinamide, and ethambutol dihydrochloride.

Drug	Water Solubility (mg/mL) at 20 °C	Highest Dose (mg)	Saturability Index	EC_50_ (mg/mL)	Taste Indexhuman	IC_50_ (mg/mL)	Taste Index BATA
Isoniazid	125 [36]	300	1	35.52	3.5	11.1	11.3
Rifampicin	0.85 [37]	600	1	2.96	0.3	1.08	0.8
Pyrazinamide	15 [38]	400 or 800	1	19.45	0.8	-	-
Ethambutol dihydrochloride	1000 [39]	400	0.4	5.52	72.5	3.78	105.8

## Supplementary Materials

The following are available online at https://www.mdpi.com/1999-4923/12/4/369/s1, Figure S1: pK_a_ values of isoniazid. Figure S2: pKa values of rifampicin. Table S1: Results of ANOVA testing to determine if sensor response for each concentration of isoniazid were significantly different to that of water (indicated by 0) and each other (*p* < 0.05). Y indicates significant difference, and N indicates no significant difference. Table S2: Results of ANOVA testing to determine if sensor response for each concentration of rifampicin were significantly different to that of water (indicated by 0) and each other (*p* < 0.05). Y indicates significant difference, and N indicates no significant difference. Table S3: Results of ANOVA testing to determine if sensor response for each concentration of pyrazinamide were significantly different to that of water (indicated by 0) and each other (*p* < 0.05). Y indicates significant difference, and N indicates no significant difference.
