# Supplementary material for: Multi-Methodological Quantitative Taste Assessment of Anti-Tuberculosis Drugs to Support the Development of Palatable Paediatric Dosage Forms
Source: Pharmaceutics. 2020 Apr 17;12(4):369. doi: 10.3390/pharmaceutics12040369 (PMC7238065; doi:10.3390/pharmaceutics12040369)
Supplement: Supplementary file 1 [file pharmaceutics-12-00369-s001.pdf]

pharmaceutics

Supplementary Materials: Multi-Methodological Quantitative Taste Assessment of Anti-Tuberculosis Drugs to Support the Development of Palatable Paediatric Dosage Forms

Alison V. Keating, Jessica Soto, Claire Forbes, Min Zhao, Duncan Q. M. Craig and Catherine Tuleu

Table S1: Results of ANOVA testing to determine if sensor response for each concentration of isoniazid were significantly different to that of water (indicated by 0) and each other ($p < 0.05$). Y indicates significant difference, N indicates no significant difference.

Concentration 1 (mM)	Concentration 2 (mM)	AC0	AN0	C00	AE1
0	9.11	Y	Y	Y	N
0	18.23	N	Y	Y	N
0	36.46	N	Y	Y	N
0	72.92	Y	Y	Y	Y
0	145.82	Y	N	Y	Y
0	291.67	Y	N	Y	Y
9.11	18.23	Y	N	Y	N
9.11	36.46	Y	N	Y	N
9.11	72.92	N	Y	Y	Y
9.11	145.84	N	N	Y	Y
9.11	291.67	N	Y	Y	Y
18.23	36.46	N	N	Y	N
18.23	72.92	Y	N	Y	Y
18.23	145.84	Y	Y	Y	Y

18.23	291.67	N	Y	Y	N
36.46	72.92	Y	N	Y	N
36.46	145.84	Y	Y	Y	Y
36.46	291.67	N	Y	Y	Y
72.92	145.84	N	N	Y	N
72.92	291.67	N	Y	N	Y
145.84	291.67	N	N	Y	N

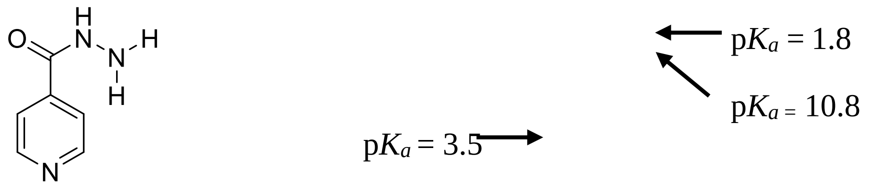

Figure S1: pK_a values of isoniazid.

Table S2: Results of ANOVA testing to determine if sensor response for each concentration of rifampicin were significantly different to that of water (indicated by 0) and each other ($p < 0.05$). Y indicates significant difference, N indicates no significant difference.

Concentration 1 (mM)	Concentration 2 (mM)	AC0	AN0	C00	AE1
0	0.24	Y	Y	Y	Y
0	0.73	Y	Y	Y	Y
0	1.22	Y	Y	Y	Y
0	1.70	Y	Y	Y	Y
0	2.19	Y	N	Y	Y
0	2.67	Y	Y	Y	Y

0.24	0.73	Y	Y	Y	Y
0.24	1.22	Y	Y	N	Y
0.24	1.70	Y	Y	N	Y
0.24	2.19	Y	Y	Y	Y
0.24	2.67	Y	Y	Y	N
0.73	1.22	N	Y	Y	Y
0.73	1.70	Y	Y	Y	Y
0.73	2.19	Y	Y	N	Y
0.73	2.67	Y	Y	Y	Y
1.22	1.70	Y	Y	N	Y
1.22	2.19	Y	Y	Y	Y
1.22	2.67	Y	Y	Y	Y
1.70	2.19	Y	Y	Y	Y
1.70	2.67	Y	Y	Y	Y
2.19	2.67	Y	Y	N	Y

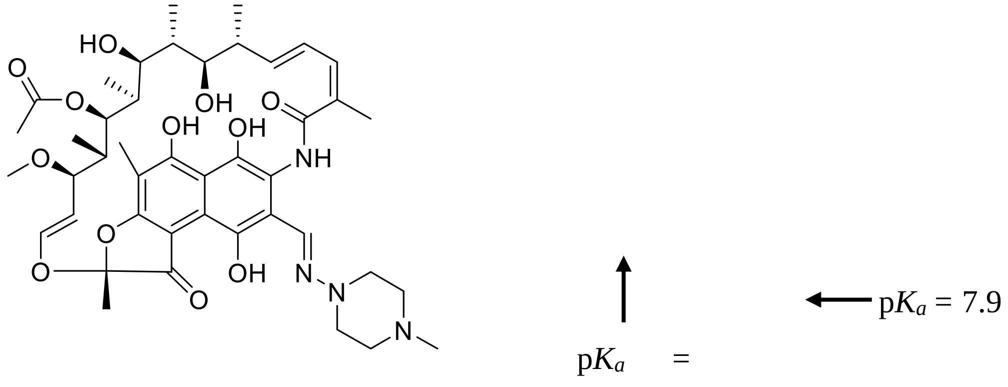

Figure S2: pK_a values of rifampicin.

Table S3: Results of ANOVA testing to determine if sensor response for each concentration of pyrazinamide were significantly different to that of water (indicated by 0) and each other ($p < 0.05$). Y indicates significant difference, N indicates no significant difference.

Concentration 1 (mM)	Concentration 2 (mM)	AC0	AN0	C00	AE1
0	3.55	Y	Y	Y	Y
0	7.11	Y	Y	Y	Y
0	14.21	Y	Y	Y	N
0	28.43	Y	Y	Y	Y
0	56.89	Y	N	Y	Y
0	113.72	Y	Y	Y	Y
3.55	7.11	Y	N	Y	Y
3.55	14.21	Y	N	Y	Y
3.55	28.43	Y	N	Y	Y
3.55	56.89	Y	Y	Y	Y
3.55	113.72	Y	Y	Y	Y
7.11	14.21	Y	N	Y	Y
7.11	28.43	Y	N	Y	Y
7.11	56.89	Y	Y	Y	Y
7.11	113.72	Y	Y	Y	Y
14.21	28.43	N	N	N	N
14.21	56.89	Y	Y	Y	Y
14.21	113.72	Y	Y	Y	Y

28.43	56.89	Y	Y	Y	N
28.43	113.72	Y	Y	N	Y
56.89	113.72	N	Y	N	Y
